# Supplementary material for: Causal Relationship between Aging and Anorexia Nervosa: A White-Matter-Microstructure-Mediated Mendelian Randomization Analysis
Source: Biomedicines. 2024 Aug 16;12(8):1874. doi: 10.3390/biomedicines12081874 (PMC11351342; doi:10.3390/biomedicines12081874)
Supplement: Supplementary file 1 [file biomedicines-12-01874-s001.zip › Supplementary Figure/Figure S4/Singe SNP plot of MR analysis for TL to ubm-b-1824.pdf]

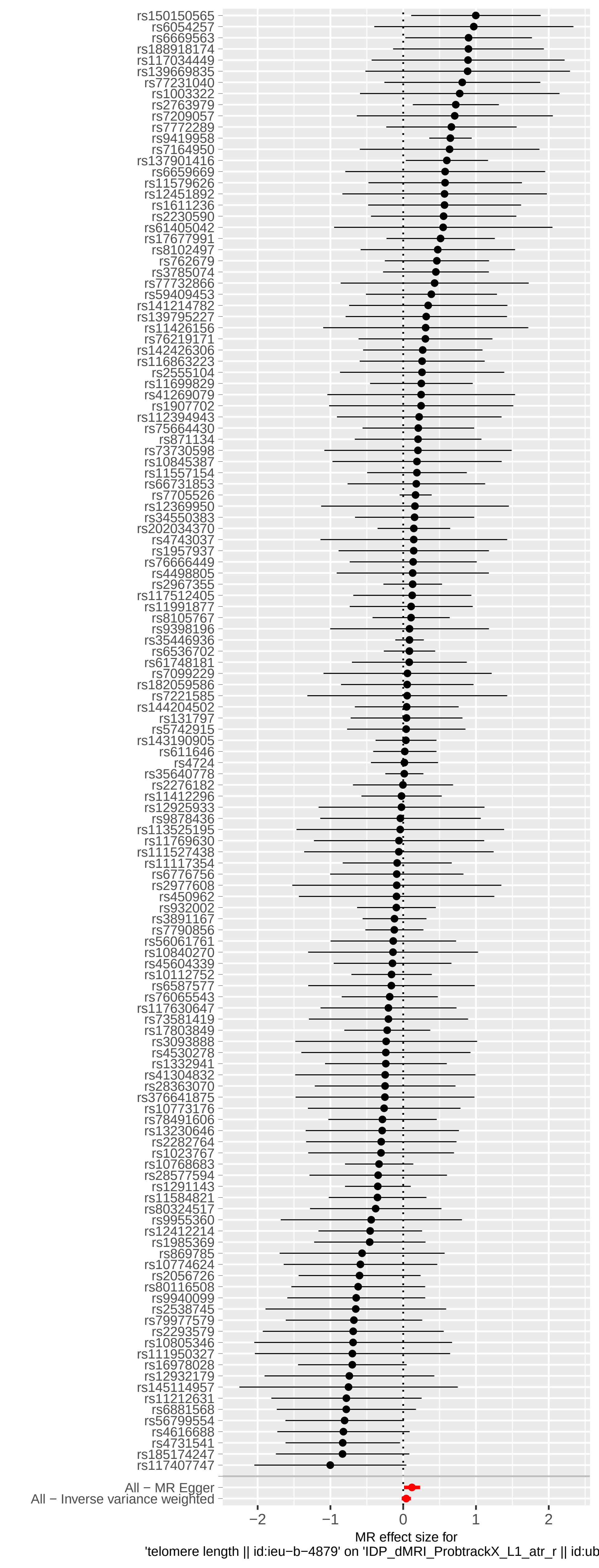
rs150150565
rs6054257
rs6669563
rs188918174
rs117034449
rs139669835
rs77231040
rs1003322
rs2763979
rs7209057
rs7772289
rs9419958
rs7164950
rs137901416
rs6659669
rs11579626
rs12451892
rs1611236
rs2230590
rs61405042
rs17677991
rs8102497
rs762679
rs3785074
rs77732866
rs59409453
rs141214782
rs139795227
rs11426156
rs76219171
rs142426306
rs116863223
rs2555104
rs11699829
rs41269079
rs1907702
rs112394943
rs75664430
rs871134
rs73730598
rs10845387
rs11557154
rs66731853
rs7705526
rs12369950
rs34550383
rs202034370
rs4743037
rs1957937
rs76666449
rs4498805
rs2967355
rs117512405
rs11991877
rs8105767
rs9398196
rs35446936
rs6536702
rs61748181
rs7099229
rs182059586
rs7221585
rs144204502
rs131797
rs5742915
rs143190905
rs611646
rs4724
rs35640778
rs2276182
rs11412296
rs12925933
rs9878436
rs113525195
rs11769630
rs111527438
rs11117354
rs6776756
rs2977608
rs450962
rs932002
rs3891167
rs7790856
rs56061761
rs10840270
rs45604339
rs10112752
rs6587577
rs76065543
rs117630647
rs73581419
rs17803849
rs3093888
rs4530278
rs1332941
rs41304832
rs28363070
rs376641875
rs10773176
rs78491606
rs13230646
rs2282764
rs1023767
rs10768683
rs28577594
rs1291143
rs11584821
rs80324517
rs9955360
rs12412214
rs1985369
rs869785
rs10774624
rs2056726
rs80116508
rs9940099
rs2538745
rs79977579
rs2293579
rs10805346
rs111950327
rs16978028
rs12932179
rs145114957
rs11212631
rs6881568
rs56799554
rs4616688
rs4731541
rs185174247
rs117407747
All − MR Egger
All − Inverse variance weighted
−2
−1
0
1
2
MR effect size for
'telomere length || id:ieu−b−4879' on 'IDP_dMRI_ProbtrackX_L1_atr_r || id:ub
